# Prognostic nomogram for predicting lower extremity deep venous thrombosis in ruptured intracranial aneurysm patients who underwent endovascular treatment

**DOI:** 10.3389/fneur.2023.1202076

**Published:** 2023-08-07

**Authors:** Chengwei Zhang, Jiaqian Zhu, Minghong Zhang, Ziru Yuan, Xiaoxiong Wang, Chengxing Ye, Haojie Jiang, Xiong Ye

**Affiliations:** ^1^Department of Neurosurgery, The First Affiliated Hospital of Wenzhou Medical University, Wenzhou, China; ^2^Wenzhou Medical University, Wenzhou, China

**Keywords:** aneurysmal subarachnoid hemorrhage, deep venous thrombosis, nomogram, endovascular treatment, prediction

## Abstract

**Background:**

Lower extremity deep vein thrombosis (DVT) is one of the major postoperative complications in patients with ruptured intracranial aneurysms (RIA) who underwent endovascular treatment (EVT). However, patient-specific predictive models are still lacking. This study aimed to construct and validate a nomogram model for estimating the risk of lower extremity DVT for RIA patients who underwent EVT.

**Methods:**

This cohort study enrolled 471 RIA patients who received EVT in our institution between 1 January 2020 to 4 February 2022. Perioperative information on participants is collected to develop and validate a nomogram for predicting lower extremity DVT in RIA patients after EVT. Predictive accuracy, discriminatory capability, and clinical effectiveness were evaluated by concordance index (C-index), calibration curves, and decision curve analysis.

**Result:**

Multivariate logistic regression analysis showed that age, albumin, D-dimer, GCS score, middle cerebral artery aneurysm, and delayed cerebral ischemia were independent predictors for lower extremity DVT. The nomogram for assessing individual risk of lower extremity DVT indicated good predictive accuracy in the primary cohort (c-index, 0.92) and the validation cohort (c-index, 0.85), with a wide threshold probability range (4–82%) and superior net benefit.

**Conclusion:**

The present study provided a reliable and convenient nomogram model developed with six optimal predictors to assess postoperative lower extremity DVT in RIA patients, which may benefit to strengthen the awareness of lower extremity DVT control and supply appropriate resources to forecast patients at high risk of RIA-related lower extremity DVT.

## Introduction

A ruptured intracranial aneurysm (RIA) is a severe neurologic emergency with devastating effects and unfavorable outcomes. It is demonstrated that the case fatality of RIA can be as high as 30–50% and at least 20% of those who survive are unable to regain functional independence (1–4). Since the publication of the International Subarachnoid Aneurysm Trial (ISAT) study, there has been a tendency toward endovascular treatment (EVT) of RIA as the first line of therapy for making smaller surgical incisions and giving patients a better health-related quality of life (5–7). The EVT of RIA has made huge strides but postoperative complications, especially lower extremity deep vein thrombosis (DVT), can not only cause swelling, localized pain, and varicose veins but may also lead to pulmonary embolism (PE), and is still a common and serious disease among RIA patients (8, 9). Several recent studies show that the incidence of lower extremity DVT ranges from 5 to 21%, and the mortality rate is between 9 and 19% (9–13).

Therefore, it is a very urgent problem to identify and predict RIA patients at the highest risk for lower extremity DVT as soon as possible. However, many factors that drive thrombosis in veins or affect its resolution remain unclear (14). Studies have indicated that intraparenchymal cerebral hemorrhage, motor deficit, high D-dimer level at hospitalization, and postoperative paralysis are key risk factors. Additionally, some studies found that an increased neutrophil-to-lymphocyte ratio is valuable in predicting lower extremity DVT (1, 11, 15). However, there is still a lack of an accurate and effective tool to predict the postoperative incidence of lower extremity DVT for RIA patients.

A mathematical model capable of predicting the risk of lower extremity DVT for RIA patients may be an approach to address this problem. A nomogram is a graphical depiction that presents a regression model in a friendly manner and simplifies risk assessment, providing medical staff with a user-friendly interface to map the probability of an event to individual patients, which has widely been used in intracranial aneurysms research (16–18). The purpose of this study is to develop and validate a nomogram model, thus providing a promising and facile avenue for predicting the risk of lower extremity DVT after RIA patients underwent EVT and promoting patient recovery.

## Methods

### Study population

**Figure 1:**
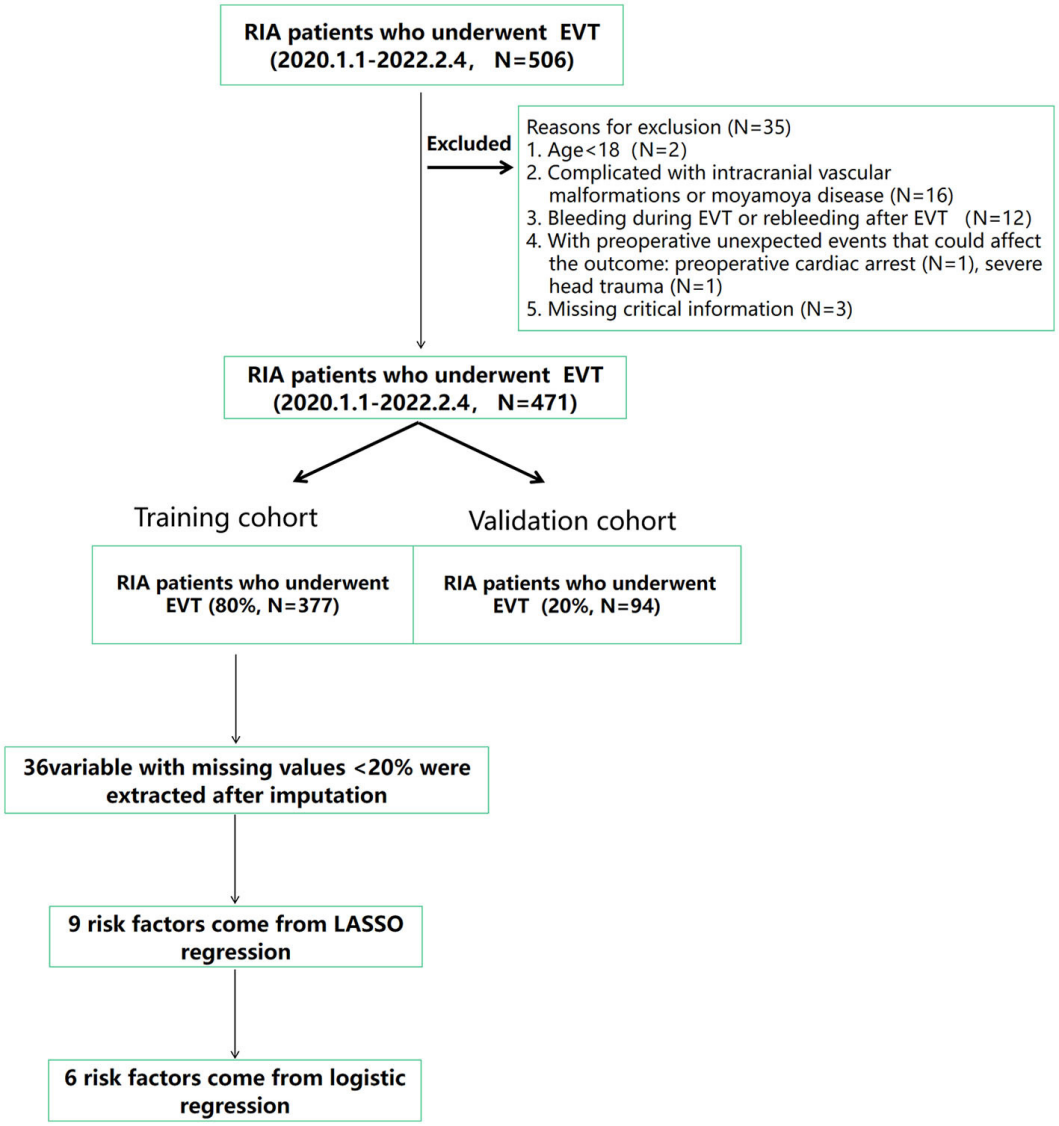
Flow diagram of the study showing the selection of RIA patients who were included in the analysis. DVT, deep venous thrombosis; EVT, endovascular treatment; RIA, ruptured intracranial aneurysms.

This is a retrospective cohort study. A total of 507 patients newly diagnosed with RIA and undergoing EVT in the First Affiliated Hospital of Wenzhou Medical University from 1 January 2020 to 4 February 2022 were included. Study samples and treatment data were retrieved from the respective surgical department databases. Patients with the following characteristics were excluded: (1) age < 18 years (*N* = 2), (2) complicated with intracranial vascular malformations or moyamoya disease (*N* = 16), (3) bleeding during EVT or rebleeding after EVT (*N* = 12), (4) patients with preoperative unexpected events that could affect the outcome: preoperative cardiac arrest (*N* = 1) and severe head trauma (*N* = 1), and (5) missing critical information (*N* = 3). Finally, a total of 471 cases were enrolled; in the enrolled patients, 377 RIA patients were included in the training cohort, and additional data were included in the validation cohort. Figure 1 shows the flow diagram of the screening process in detail. The data used in this study were approved by the Institutional Review Board of Wenzhou Medical University which waived the requirement for individual patient consent because they did not contain personal identifiers. This study follows the principles of the Declaration of Helsinki (19).

### Variable definition and data collection

RIA patients were diagnosed by biplanar digital subtraction angiography (DSA) after the diagnosis of subarachnoid hemorrhage by head computed tomography (CT) (20, 21). Our diagnosis criterion for lower extremity DVT was based on the observation of lower limb deep vein intravascular shadows by Doppler ultrasound (DUS) that included B-mode imaging and color Doppler flow imaging with or without probe compression within 2 weeks of EVT treatment (22, 23). Considering the high incidence of lower extremity DVT, each patient needs to be examined by DUS every week after admission to our institution. Other variables include gender, age, height, underlying diseases (hypertension, diabetes, coronary heart disease, and infection), the location of the aneurysm [anterior communicating artery (ACoA), posterior communicating artery (PCoA), internal carotid artery (ICA), vertebrobasilar aneurysm (VBA)], stent-assisted EVT, ventricular drainage, smoking, modified Fisher scale, Hunt-Hess grades, World Federation of Neurosurgical Societies (WFNS) grade, Glasgow Coma Scale (GCS), and presence or absence of motor deficits on admission, laboratory examination on admission (total cholesterol, uric acid, triglyceride, glucose, albumin, D-dimer, C-reactive protein (CRP), mean corpuscular volume (MCV), hemoglobin, lymphocyte count, monocyte count, neutrophil counts, and platelet count), mechanical ventilation time after EVT, and delayed cerebral ischemia (DCI) after EVT.

### Statistical analysis

SPSS software version 25.0 and R software version 4.0.2 were used for statistical analysis. Continuous variables with normal distribution were expressed as mean and standard deviation (SD), and differences between groups were compared by an independent sample *t*-test. Continuous variables that were not normally distributed were represented by median and range, and differences between groups were compared using the Mann–Whitney U-test. Categorical variables were presented as frequencies or percentages, and the chi-square tests were used to compare differences between groups. The least absolute contraction and selection operator (LASSO) regression model was used to deal with the collinearity problem of candidate variables to select the optimal predictor variables. A logistic regression analysis was used to estimate univariate and multivariate odds ratios and 95% confidence intervals. A *P*-value of <0.05 was statistically significant, and the C-index was used to evaluate the discrimination of the model. Clinical utility and net benefits were determined by decision curve analysis (DCA) (24). In total, 1,000 bootstrap re-samples were used for external validation, and the relative corrected C-index was calculated to ensure the stability of the nomogram in the validation cohort.

## Result

### Population characteristics

Table 1 shows the clinical characteristics of the study population. A total of 471 RIA patients who underwent EVT were enrolled in this study, separated by the training cohort (377 patients, from 1 January 2020 to 16 November 2020) and validation cohort (94 patients, from 17 August 2021 to 4 February 2022). Patients' age ranged from 18 to 89 years (54.0 ± 15.5). Among the primary cohort patients, 42 patients had lower extremity DVT, leading to an incidence rate of 8.9%. Demographics and clinical and laboratory characteristics for both cohorts are shown in Table 1. No significant differences were noted between the cohorts.

**Table 1:** Characteristics of the study population.

**Variable**	**Total (*n* = 471)**	**Training cohort (*n* = 377)**	**Validation cohort (*n* = 94)**	***p***
Age, median [IQR]	56.0 [49.0, 66.0]	57.0 [49.0, 67.0]	53.0 [48.0, 64.0]	0.083
Gender (male), *n* (%)	158 (33.5)	124 (32.9)	34 (36.2)	0.547
Smoking, *n* (%)	66 (14.0)	50 (13.3)	16 (17.0)	0.348
Hypertension, *n* (%)	249 (52.9)	203 (53.846)	46 (48.936)	0.394
Diabetes, *n* (%)	34 (7.2)	25 (6.631)	9 (9.574)	0.324
Coronary heart disease, *n* (%)	13 (2.8)	11 (2.918)	2 (2.128)	0.676
Infection, *n* (%)	84 (17.8)	69 (18.302)	15 (15.957)	0.595
DCI, *n* (%)	27 (5.7)	23 (6.101)	4 (4.255)	0.491
Timing to DCI (day), mean (±SD)	6.0 ± 2.0	5.9 ± 2.0	6.3 ± 1.9	0.768
ACoA aneurysm, *n* (%)	142 (30.1)	121 (32.1)	21 (22.3)	0.065
PCoA aneurysm, *n* (%)	108 (22.9)	85 (22.5)	23 (24.5)	0.692
ICA aneurysm, *n* (%)	139 (29.5)	112 (29.7)	27 (28.7)	0.851
MCA aneurysm, *n* (%)	17.8 (84)	65 (17.2)	19 (20.2)	0.501
VBA aneurysm, *n* (%)	20 (4.2)	14 (3.7)	6 (6.4)	0.251
External ventricular drainage, *n* (%)	55 (11.7)	46 (12.2)	9 (9.574)	0.478
Stent-assisted EVT, *n* (%)	235 (49.9)	196 (51.9)	39 (41.4)	0.069
Height (cm), median [IQR]	160.0 [156.0, 165.0]	160.0 [156.0, 165.0]	160.0 [155.0, 167.0]	0.929
GCS, median [IQR]	15.0 [15.0, 15.0]	15.0 [15.0, 15.0]	15.0 [15.0, 15.0]	0.924
WFNS grade, median [IQR]	1.0 [1.0, 2.0]	1.0 [1.0, 2.0]	1.0 [1.0, 1.0]	0.171
Modified Fisher Scale, median [IQR]	2.0 [1.0, 2.0]	2.0 [1.0, 3.0]	2.0 [1.0, 2.0]	0.500
Hunt-Hess grades, median [IQR]	2.0 [1.0, 2.0]	2.0 [1.0, 2.0]	2.0 [2.0, 2.0]	0.066
Motordeficit, *n* (%)	84 (17.8)	68 (18.0)	16 (17.0)	0.818
Mechanical ventilation time, median [IQR]	0.0 [0.0, 1.0]	0.0 [0.0, 0.0]	0.0 [0.0, 1.0]	0.068
Hemoglobin (g/L), median [IQR]	132.0 [120.0, 142.0]	132.0 [119.0, 142.0]	134.0 [123.0, 144.0]	0.125
MCV, (fl), mean (±SD)	89.1 ± 9.4	88.8 ± 10.2	90.1 ± 4.5	0.274
Lymphocyte count (^*^10^9/^L), median [IQR]	1.1 [0.9, 1.5]	1.1 [0.8, 1.6]	1.2 [0.9, 1.5]	0.831
Monocyte count (^*^10^9/^L), median [IQR]	0.5 [0.3, 0.7]	0.5 [0.3, 0.7]	0.5 [0.3, 0.7]	0.362
Neutrophil counts (^*^10^9/^L), median [IQR]	9.7 [7.0, 12.5]	9.6 [7.0, 12.4]	10.1 [7.4, 13.0]	0.317
NLR, median [IQR]	24.5 [17.7, 37.2]	25.8 [17.7, 37.9]	21.9 [16.0, 34.8]	0.107
Platelet count (^*^10^9/^L), median [IQR]	213.0 [175.0, 258.0]	213.0 [175.0, 256.0]	211.000 [175.0, 262.0]	0.733
Total cholesterol (μmlo^/^L), mean (±SD)	4.9 ± 1.2	5.0 ± 1.2	5.0 ± 1.0	0.895
Uric acid (μmlo^/^L), median [IQR]	242.0 [176.0, 302.0]	245.0 [176.0, 302.0]	228.0 [176.0, 291.0]	0.365
Triglyceride (μmlo^/^L), median [IQR]	1.1 [0.8, 1.7]	1.1 [0.8, 1.6]	1.1 [0.8, 1.7]	0.881
Glucose (mmlo^/^L), median [IQR]	6.5 [5.4, 7.7]	6.4 [5.4, 7.6]	6.5 [5.1, 8.1]	0.772
Albumin (g/L), median [IQR]	39.1 [36.9, 41.5]	39.1 [36.9, 41.5]	39.0 [36.9, 41.7]	0.692
D-dimerlevel (μg/mL), median [IQR]	1.1 [0.5, 2.5]	1.1 [0.5, 2.5]	1.3 [0.6, 2.8]	0.278
CRP (mg/L),median [IQR]	5.6 [2.3, 15.5]	5.6 [2.3, 15.7]	6.4 [1.9, 14.6]	0.587
DVT, *n* (%)	42 (8.9)	37 (9.8)	5 (5.3)	0.171
Timing to DVT diagnosis (day), mean (±SD)	10.0 ± 4.6	9.8 ± 4.4	11.0 ± 6.3	0.602
Mortality within 30 days of onset, *n* (%)	18	14	4	0.806

ACoA, anterior communicating artery; PCoA, posterior communicating artery; ICA, internal carotid artery; MCA, middle cerebral artery; VBA, vertebrobasilar aneurysm; CRP, C-reactive protein; MCV, mean corpuscular volume; GCS, Glasgow Coma Scale; WFNS, World Federation of Neurosurgical Societies; DCI, delayed cerebral ischemia; NLR, neutrophil-to-lymphocyte ratio.

### Selected predictors

**Figure 2:**
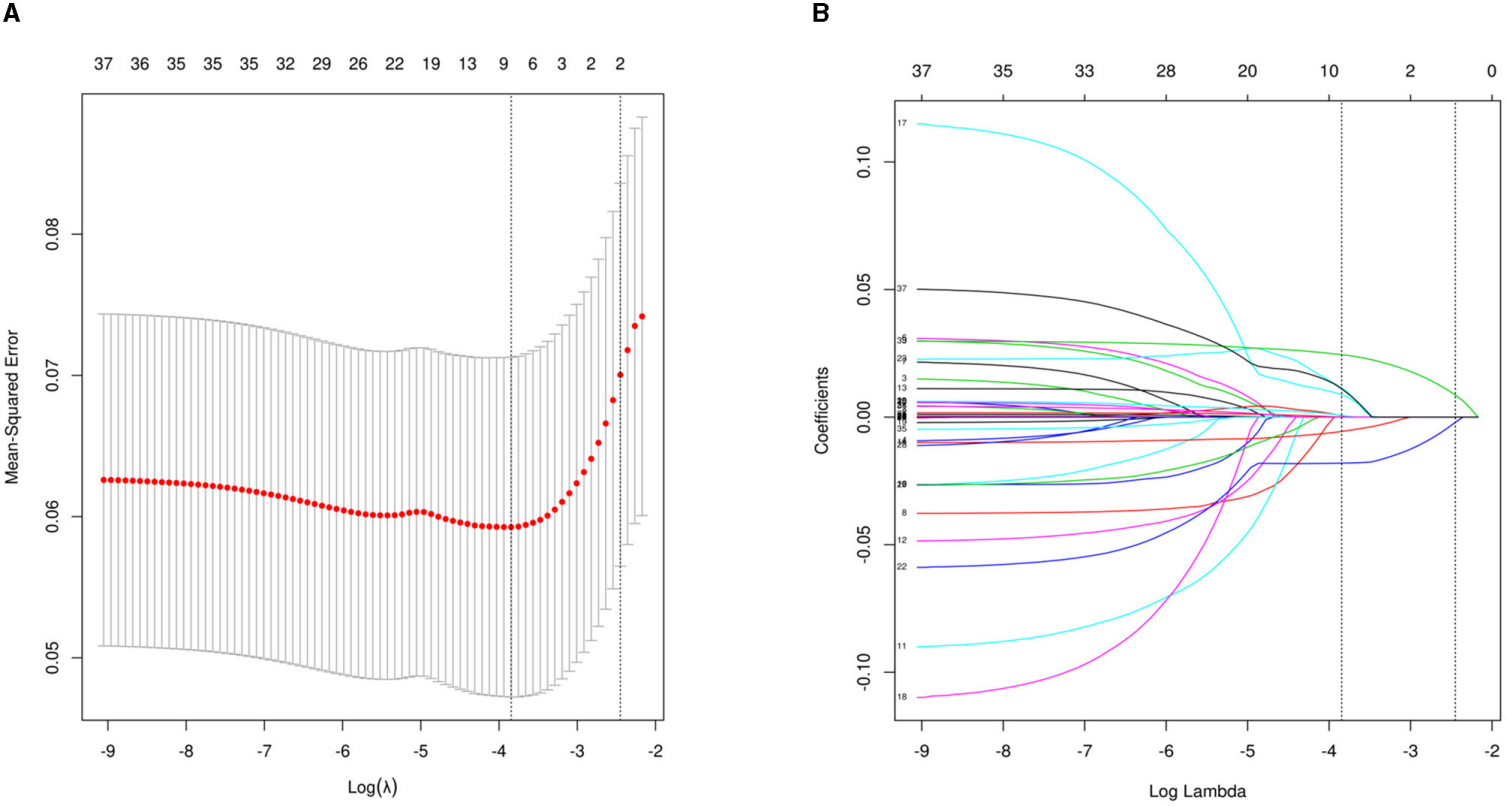
Perioperative variable selection using a LASSO logistic regression model. **(A)** Dotted vertical lines were depicted at the optimal values by using the minimum criteria (lambda.min) and 1 SE of the minimum criteria (lambda.1se). **(B)** LASSO coefficient profile of 36 variables. The model achieves optimality at lambda.1SE, where variables with nonzero coefficients are screened out as potential predictors, effectively reducing the number of influencing factors from 36 to 9.

Of the 36 variables with missing values, <20% was extracted after interpolation, and nine potential predictors were finally screened from the LASSO regression analysis (Figure 2). Inclusion of these nine variables in a logistic regression model resulted in six variables that were independently statistically significant predictors of critical illness and were included in the risk score (Table 2). These variables include age (OR, 1.07; 95% CI, 1.02–1.11; *P* = 0.003), albumin level (OR, 0.88; 95% CI, 0.81–0.96; *P* = 0.005), D-dimer level (OR, 1.24; 95% CI, 1.12–1.39; *P* < 0.001), GCS score (OR, 0.72; 95% CI, 0.63–0.81; *P* < 0.001), middle cerebral artery (MCA) aneurysm (OR, 5.67; 95% CI, 1.95–16.94; *P* = 0.001), and DCI (OR, 3.79; 95% CI, 1.03–13.244; *P* = 0.039).

**Table 2:** Multivariable logistic regression model for predicting lower extremity DVT in RIA patients.

**Predictor**	**β**	**SE**	***p*-value**	**Odds ratio (95% CI)**
Intercept	1.57	2.13	0.460	4.80 (0.07–305.42)
Age, per y	0.06	0.02	0.003	1.06 (1.02–1.11)
GCS, per score	−0.33	0.06	< 0.001	0.72 (0.63–0.81)
Albumin, g/L	−0.12	0.04	0.005	0.88 (0.81–0.96)
D-dimer level,	0.22	0.05	< 0.001	1.24 (1.12–1.39)
MCA aneurysm (yes vs. no)	1.74	0.55	0.001	5.67 (1.95–16.94)
DCI (yes vs. no)	1.33	0.65	0.039	3.79 (1.03–13.244)

GCS, Glasgow Coma Scale; MCA, middle cerebral artery; DCI, delayed cerebral ischemia. RIA, ruptured intracranial aneurysms.

### Construction and validation of the nomogram

**Figure 3:**
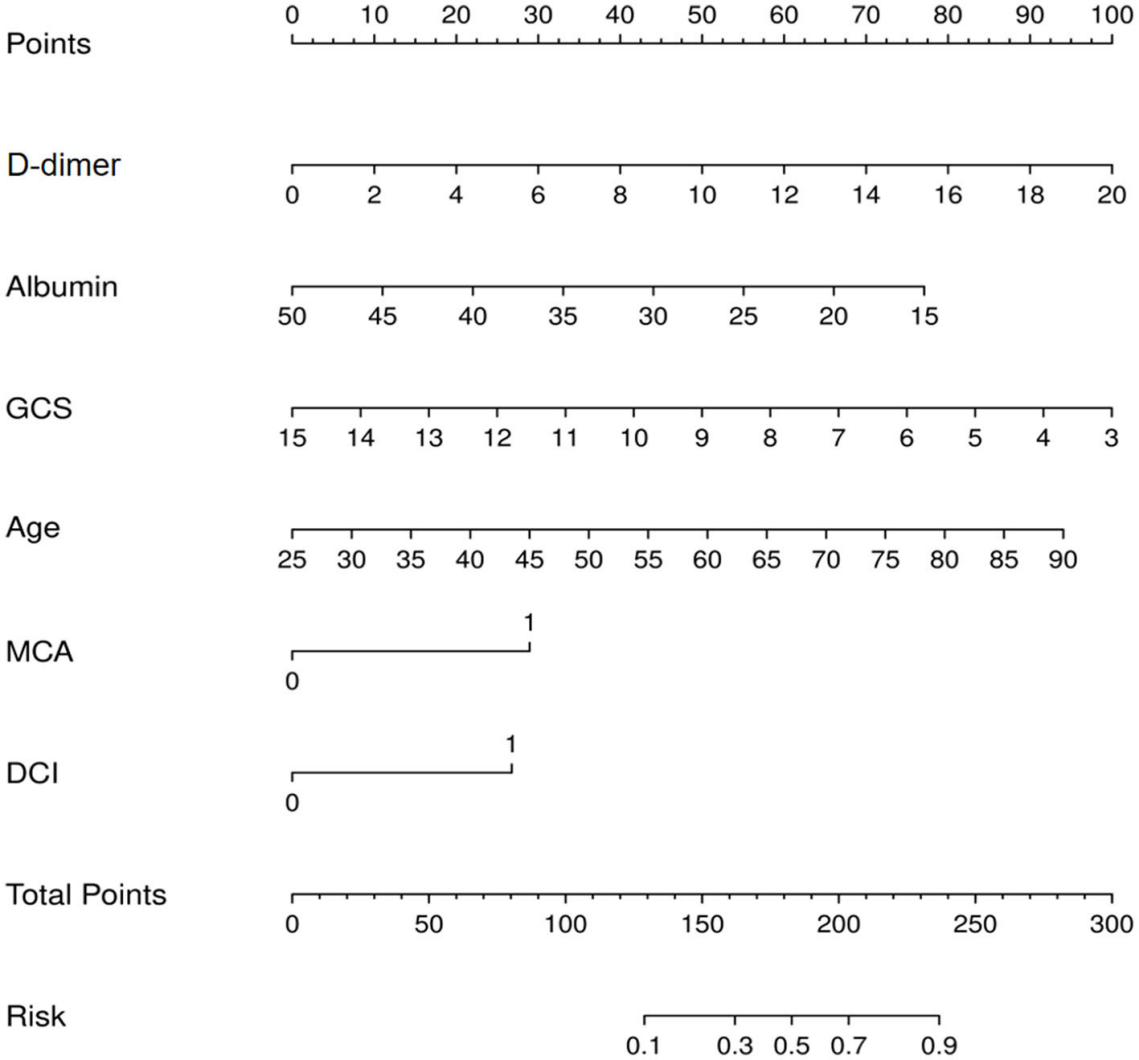
Nomogram for prediction of lower extremity deep vein thrombosis (DVT) in ruptured intracranial aneurysms (RIA) patients who underwent endovascular treatment (EVT).

**Figure 4:**
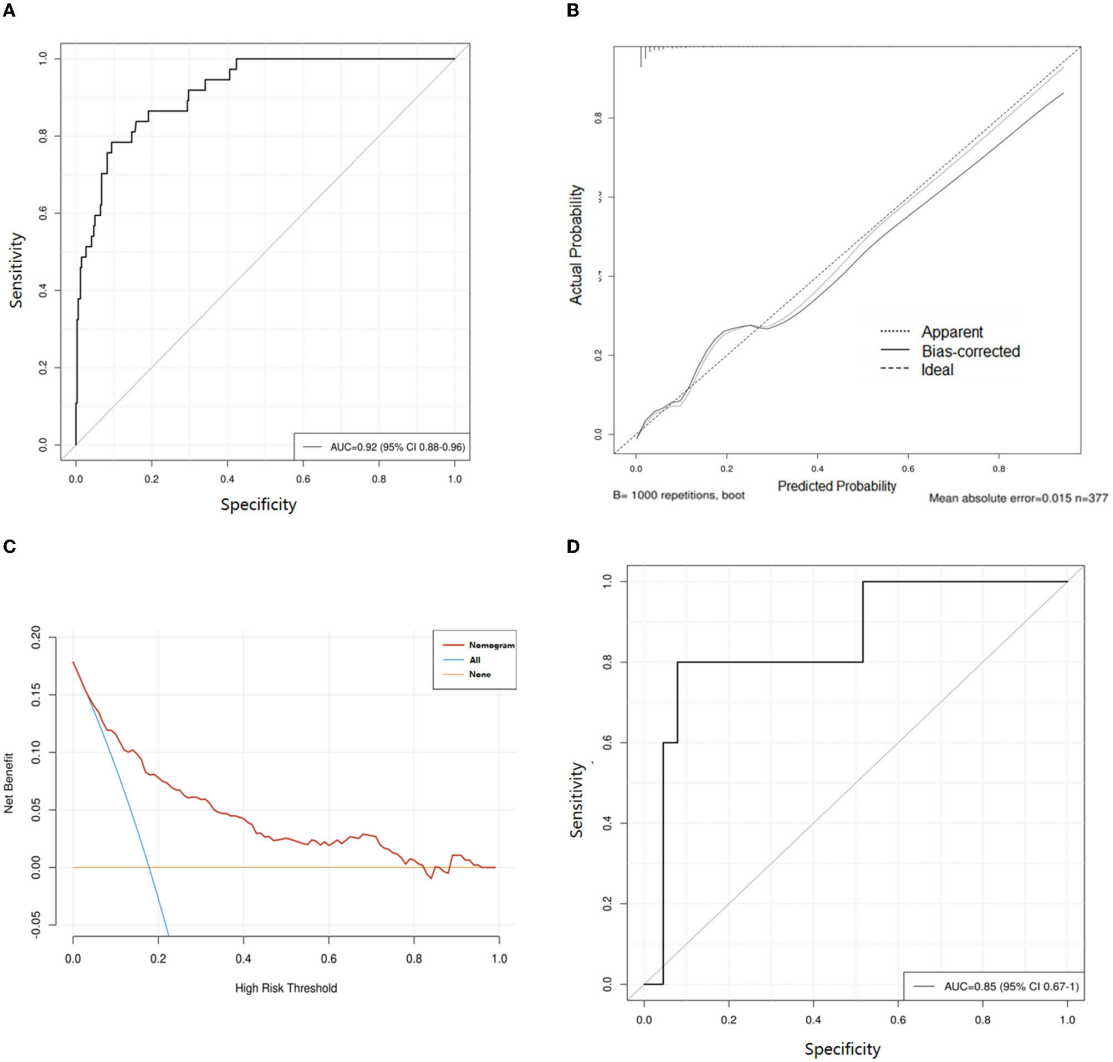
Evaluation of the nomogram for prediction of lower extremity deep vein thrombosis (DVT) in ruptured intracranial aneurysms (RIA) patients who underwent endovascular treatment (EVT). **(A)** Receiver operating characteristic curves of the training cohort. **(B)** Calibration curves of the DVT predictive nomogram in the training cohort. **(C)** Decision curve analysis for assessing the clinical usefulness of the low extremity DVT nomogram. **(D)** Receiver operating characteristic curves of validation cohort. AUC, area under the receiver operating characteristic curve.

The nomogram for predicting lower extremity DVT probability in RIA patients who underwent EVT was shown in Figure 3. In this model, the D-dimer level and GCS score exhibited the greatest influence on lower extremity DVT, followed by age, albumin level, MCA aneurysm, and DCI, respectively. Quantified by the C-index, model discrimination was 0.92, demonstrating that the prognostic model effectively predicted lower extremity DVT (Figure 4A). The calibration plot (Figure 4B) indicated good consistency between the predictive risk of lower extremity DVT and the observed real risk. Based on the net benefit and threshold probabilities, the clinical value of the nomogram was evaluated using a decision curve analysis. As for lower extremity DVT of RIA patients, the graph (Figure 4C) indicated that the nomogram threshold probability analysis had a preferable net benefit (with a wide range of 4–82%). We externally verified the nomogram generated in the training cohort with the aim of confirming the stability of the model. The validation cohort was made up of 94 RIA patents from 17 November 2020 to 4 February 2022 when applied to a validation cohort with a C-index of 0.85 (Figure 4D).

## Discussion

In this retrospective study, we developed and validated a clinical risk score and a nomogram to predict the development of lower extremity DVT among RIA patients who underwent EVT treatment. The performance of this risk score was satisfactory with accuracy based on C-indexes in the development cohorts of 0.92 and validation cohorts of 0.85, which showed that it was discriminatory and well-calibrated, and external validation showed satisfactory accuracy and generalizability. Moreover, the logistic regression analysis demonstrated that age, GCS score, MCA aneurysm, DCI, albumin, and D-dimer were the best predictors of low extremity DVT.

Due to the current advances in antithrombotic therapy and thrombus monitoring, the death of aSAH patients caused by DVT has not occurred in our center since 2020. DVT mainly leads to the length of hospital stay and increases the medical cost of patients, and anticoagulant therapy may increase the risk of bleeding in patients without surgical treatment (25, 26). This suggests that we still need to recognize the risk factors of DVT and actively intervene in the treatment.

It is almost universally accepted that there is an association between age and lower extremity DVT. The older the patient was, the worse his/her functional status became, and the more likely he/she was to present in poor condition following aSAH, such as the activation of coagulation and decreased muscle strength (26–29). Moreover, our research provides several new insights. First of all, our study revealed that a low GCS score, MCA aneurysm, and DCI were important predictors for lower extremity DVT. The risk factor of a low GCS score has been reported in many studies (30, 31), but MCA aneurysm and DCI have not been reported as independent risk factors for lower extremity DVT. This may be because a ruptured MCA aneurysm had the highest rate of parenchymatous hematoma, which reaches 40–50% in all kinds of intracranial aneurysms (32, 33). Hematomas destroy brain parenchyma and lead to limb disability (34, 35). Moreover, when embolizing an MCA aneurysm, the coil may affect the parent artery blood flow, which may result in the ischemia of the motor cortex and also lead to limb disability (36). DCI is a common postoperative complication affecting 20–30% of patients with RIA, which aggravates the patient's disturbance of consciousness and low limb disability (37). Above all, patients with advanced age, low GCS score, MCA aneurysm, and DCI often have poor limb function, especially the reduction of lower limb activity. Those all lead to reduced muscle contraction in the lower extremities and slower venous blood flow, which increases the likelihood of low extremity DVT (30).

Our study also confirmed a significant correlation between the level of D-dimer at admission and the development of lower extremity DVT. Abnormally elevated D-dimer levels often indicate a high likelihood of thrombosis (38). However, any process that increases fibrin production or breakdown also increases D-dimer levels, including pregnancy, inflammation, cancer, and surgery. This results in a high false-positive probability and low specificity of D-dimer in predicting. Therefore, we should dynamically detect and evaluate those patients' D-dimer levels to monitor the occurrence of DVT at an earlier stage (30).

Finally, our study also found that hypoalbuminemia was significantly associated with the occurrence of lower extremity DVT, which has been reported in the prediction of lower extremity DVT in other diseases (39, 40). This may be due to the decrease of plasma colloid osmotic pressure in patients with hypoalbuminemia, which easily leads to brain edema (41) and neurological dysfunction, thus leading to the reduction of lower limb activity and being more prone to lower extremity DVT. In addition, patients with hypoalbuminemia are more likely to have various infections after surgery (42). Postoperative infection often leads to a prolonged hospital stay and decreased activity willingness of patients, thus leading to an increased risk of lower extremity DVT (30).

In conclusion, our model can be used to assess the risk of lower extremity DVT during hospitalization for RIA patients who underwent EVT. Previous research had identified a wide heterogeneity of prophylactic protocols in neurosurgery, and any prophylaxis for DVT and PE should be individualized with careful evaluation of the benefits vs. risks (25). Our model not only incorporated age, GCS, and D-dimer, which were widely reported predictors of DVT but also identified the unique and indispensable role of DCI, albumin level, and MCA aneurysm in predicting DVT in patients with RIA. We need to pay more attention to patients at high risk of lower extremity DVT according to the score and take corresponding measures to reduce the braking time and encourage patients to get out of bed early while ensuring their safety. In addition, Chibbaro et al. (43) provided evidence supporting the safe and effective implementation of prophylactic protocols (mechanical +/- pharmacological) during the preoperative period and continued perioperatively and postoperatively (depending on the risk profile of the individual patient) with a remarkable reduction in the incidence of DVT and PE and without any increase in the risk of postoperative intracranial bleeding. Therefore, pharmacological prevention should be prioritized for RIA patients at high risk of DVT. In patients with temporary immobilization or loss of motor ability, lower limbs should be elevated, leg massage should be performed, pharmacological prevention or mechanical thrombus prevention should be performed using progressive pressure socks, intermittent pneumatic compression (IPC), etc. (44, 45). At the same time, the frequency of lower limb venous ultrasound screening should be increased to discover lower extremity DVT earlier (46).

This study has some limitations. First, the sample of participants was limited, and the study was limited by a lack of information on sufficient variables. In addition, our retrospective study is a single-center study with an unrepresentative sample, which provide only Level of Evidence 4 according to the Oxford Centre for Evidence-Based Medicine and may not be generalizable to other centers. Moreover, our data are missing intracranial hematoma volume and postoperative anticoagulation in patients with RIA. These data are critical for DVT prediction. However, these data were not included in the analysis because of the difficulties in obtaining them in our hospital and the differences in the type of prophylactic protocol and its duration, which need to be further improved in follow-up studies. Finally, due to inconsistent methodological descriptions of risk factors in included studies, we performed only systematic reviews rather than meta-analyses, so the dimensions of association of these risk factors with RIA-related lower extremity DVT are largely unclear. Therefore, these results may be interpreted with caution, and further research on these important risk factors should be warranted, which could help to ameliorate or control key risk factors for RIA-related low extremity DVT.

## Conclusion

Our study found that D-dimer level, age, albumin, and GCS score were reliable predictors of DVT. The discrimination, accuracy, clinical effectiveness, and external verification of the prognostic nomogram developed in this study led to satisfactory performance for predicting lower extremity DVT. The finding can help medical staff identify patients at high risk of lower extremity DVT for RIA patients who underwent EVT, so targeted interventions can be administered to decrease the occurrence of lower extremity DVT. Further studies that take other clinically relevant variables into account will refine the nomogram.

## Data availability statement

The raw data supporting the conclusions of this article will be made available by the authors, without undue reservation.

## Ethics statement

Ethical review and approval was not required for the study on human participants in accordance with the local legislation and institutional requirements. Written informed consent for participation was not required for this study in accordance with the national legislation and the institutional requirements.

## Author contributions

JZ, XY, and CZ: conception, design, and revision of the article. XY, MZ, ZY, CY, and XW: data collection. HJ, XY, JZ, CZ, and MZ: analysis and drafting of the article. JZ, CZ, MZ, ZY, CY, XW, HJ, and XY: contributed to the article and approved the final version.
